# Metallic Aluminum Suboxides with Ultrahigh Electrical Conductivity at High Pressure

**DOI:** 10.34133/2022/9798758

**Published:** 2022-08-28

**Authors:** Tianheng Huang, Cong Liu, Junjie Wang, Shuning Pan, Yu Han, Chris J. Pickard, Ravit Helled, Hui-Tian Wang, Dingyu Xing, Jian Sun

**Affiliations:** ^1^National Laboratory of Solid State Microstructures, School of Physics, And Collaborative Innovation Center of Advanced Microstructures, Nanjing University, Nanjing 210093, China; ^2^Department of Materials Science & Metallurgy, University of Cambridge, 27 Charles Babbage Road, Cambridge CB3 0FS, UK; ^3^Advanced Institute for Materials Research, Tohoku University, 2-1-1 Katahira, Aoba, Sendai 980-8577, Japan; ^4^Institute for Computational Science, Center for Theoretical Astrophysics & Cosmology, University of Zurich, Switzerland

## Abstract

Aluminum, as the most abundant metallic elemental content in the Earth's crust, usually exists in the form of alumina (Al_2_O_3_). However, the oxidation state of aluminum and the crystal structures of aluminum oxides in the pressure range of planetary interiors are not well established. Here, we predicted two aluminum suboxides (Al_2_O, AlO) and two superoxides (Al_4_O_7_, AlO_3_) with uncommon stoichiometries at high pressures using first-principle calculations and crystal structure prediction methods. We find that the *P4/nmm* Al_2_O becomes stable above ~765 GPa and may survive in the deep mantles or cores of giant planets such as Neptune. Interestingly, the Al_2_O and AlO are metallic and have electride features, in which some electrons are localized in the interstitials between atoms. We find that Al_2_O has an electrical conductivity one order of magnitude higher than that of iron under the same pressure-temperature conditions, which may influence the total conductivity of giant planets. Our findings enrich the high-pressure phase diagram of aluminum oxides and improve our understanding of the interior structure of giant planets.

## 1. Introduction

Aluminum oxide is one of the most abundant substances in the mantle and core of the planets [1–5]. As for the other basic constituents of planets, such as iron [6, 7], silica [8], and water [9], studying aluminum oxide and its high pressure properties is essential for us to understand the structure, formation, and evolution of planets [10–12]. As the most common aluminum oxide, alumina (Al_2_O_3_) has high hardness, good thermal, and dielectric properties, which makes it an important industrial raw material for abradant and refractory material, etc. As a window material in shock-wave experiments and one of the major components in the mantle of Earth, it is also of great importance in both high-pressure technology [13] and geophysics [3]. The structural phase transitions and chemical stability of alumina directly affect the properties of planetary cores, such as the equation of states, thermoelastic properties, electrical conductivity, and oxidation. High pressure investigations, both theoretical and experimental, have provided a complex phase diagram for alumina [1–5, 14, 15]. A sequence of pressure-induced phase transitions in alumina emerge in turn: corundum ($R\bar{3}c$) → Rh_2_O_3_-type (*Pbcn*) → CaIrO_3_-type (*Cmcm*) → U_2_S_3_-type (*Pnma*). Meanwhile, two other stable Al-O compounds (AlO_2_ and Al_4_O_7_) were predicted by a first-principle study under high pressure [16]. Overall, all of these aforementioned aluminum oxides are insulating with a wide band gap.

While the possible crystalline structures of alumina have been extensively investigated over the pressure range of Earth's mantle and core [1–5, 14, 15], we still have a limited knowledge on the compounds, and structures of aluminum oxides can form at more extreme conditions, especially in the interior of giant planets such as Jupiter, Saturn, Uranus, and Neptune [11, 12, 17], where much higher temperatures and pressures exist. Current observations of Uranus and Neptune are limited, and different models have been developed to fit these data, including the ice-dominated and rock-dominated models. Comparing to the ice-dominated models, the rock-dominated models provide a simple explanation for the formation of the Neptune [18]. However, there is still a remaining issue that the formation of planetary dynamos [10, 19] requires electrically conductive materials. Along this line, it is reported that the magma ocean in super-Earths may contribute to the magnetic field if its electrical conductivity is sufficiently high. For instance, several common mantle compounds such as silicates [20] and alumina [21] have already been shown that their electrical conductivities enlarge significantly after melting at extremely high temperatures. In addition, shock-wave experiments on silica show that it may become conductive at more than 500 GPa and 9000 K [8].

Apart from the mantle compounds, recent shock compression experiments have shown that water and ammonia become ionically conducting under high pressures present in the dynamo generation region of ice giants [22, 23]. Additionally, the conductivity of hydrogen-water mixtures is also expected to increase rapidly with depth in the outer layers of ice giants [24], where the generation of secondary magnetic fields spatially correlated with zonal winds might shed light onto the electrical conductivity profiles of solar system giants in general [25, 26]. Nevertheless, the compositional gradients and thus the electrical conductivity profiles of ice giants are still unclear. Therefore, as one of the important components of the rocky core and/or mantle of planets, searching for new structures of aluminum oxides could enhance our understanding of the electrical conductivity of giant planets.

To enrich our understanding of the physical properties of the planetary interior, we have systematically explored crystalline structures of alumina and other possible stoichiometries of the Al-O system in the pressure range expected in planetary cores using crystal structure prediction methods and first-principle calculations. Here, we report the prediction of several new aluminum suboxides and superoxides with different stoichiometries, including Al_2_O, AlO, Al_4_O_7_, and AlO_3_, together with a new high pressure phase of Al_2_O_3_. Most importantly, two of these new aluminum oxides, Al_2_O and AlO, are metallic. We find that the electrical conductivity of Al_2_O is higher than that of hcp iron under the pressure and temperature condition near the Neptune's core mantle boundary (if such a boundary exists), indicating that it can affect the planetary electrical conductivity.

The high-pressure crystal structure searching of Al-O system was performed with MAGUS [27] (machine learning and graph theory assisted universal structure searcher), which is accelerated by the employment of Bayesian optimization and graph theory [28]. This method has been successfully applied in many systems under high pressure, such as compounds inside planets [29–32]. In addition, we cross checked the searching results with AIRSS [33, 34] combined with CASTEP [35]. DFT calculations were performed using the Vienna *Ab initio* simulation package (VASP) [36], together with the projection-augmented wave (PAW) method [37]. *Ab initio* molecular dynamics simulations were performed with NVT and NPT ensembles using cubic supercells and periodic boundary conditions. The ionic temperature was controlled with a Nosé-Hoover thermostat [38, 39]. Simulations in NVT ensemble ran for 10 ps with ionic time steps of 1 fs, and 10 configurations were extracted separately in time by 0.8 ps in the last 8 ps, guaranteeing their statistical irrelevance. We took the average of the conductivity of these ionic configurations as the electrical conductivity of the system. All the electrical conductivities were calculated using the Kubo-Greenwood formula, as implemented in the Kg4vasp code [40, 41]. More details about the method can be found in the Supplemental Material.

**Figure 1:**
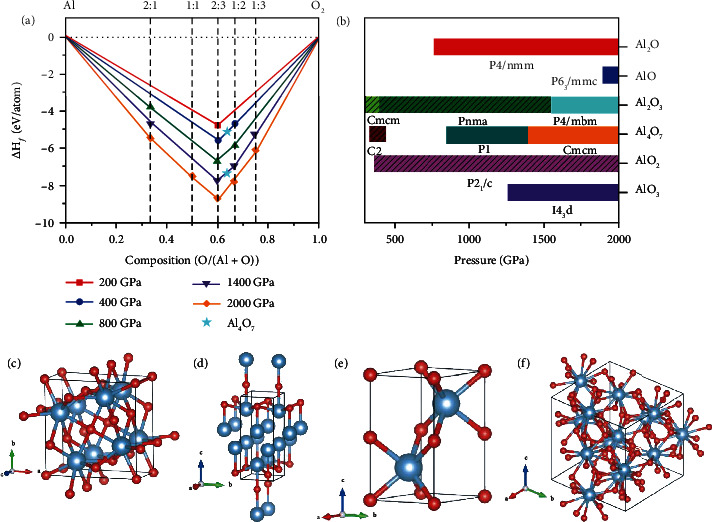
Energetics and crystal structures of the most stable compounds of the Al-O system at 200–2000 GPa. (a) Convex hulls for formation enthalpies relative to the most stable phases of pure Al [51, 52] and O_2_ [53] at different pressures. The cyan star represents Al_4_O_7_ crystals with three different structures. The C2 Al_4_O_7_ is stable only between 330 and 450 GPa, while the $P\bar{1}$ Al_4_O_7_ and the *Cmcm* Al_4_O_7_ become stable successively from 850 to 1500 to 2000 GPa. (b) Pressure-composition phase diagram of the aluminum oxides in the pressure range of 300-2000 GPa, the structures found previously are marked with pattern. The crystal structures of the *P4/mbm* Al_2_O_3_ at 1600 GPa (c), the *P4/nmm* Al_2_O at 800 GPa (d), the *P6_3_/mmc* AlO at 2000 GPa (e), and the $I{\bar{4}}_{3}d$ AlO_3_ at 1300 GPa (f), respectively. The red and silver spheres denote oxygen and aluminum atoms, respectively.

We have searched extensively for possible stoichiometries in the Al-O system in the pressure up to 2000 GPa, a pressure that can be achieved in shock-wave experiments [42]. The results are summarized in Figures 1(a) and 1(b). For Al_2_O_3_, we found a tetragonal structure with *P4/mbm* symmetry, which extends our knowledge of the structure of alumina at terapascal pressures. Enthalpy calculations show that this *P4/mbm* structure is more stable than the U_2_S_3_-type alumina above 1560 GPa, as shown in Fig. S1(a). Phonon calculations demonstrate that there are no imaginary modes at 1600 GPa, see in Fig. S4(a), confirming the robust dynamic stability of this *P4/mbm* phase under extreme pressure. In contrast to previously reported alumina structures [5], this *P4/mbm* phase does not adopt mixed coordination numbers. While the *P4/mbm* phase shares the similar Al atoms lattice with the U_2_S_3_-type phase, both the average Al_1_-O/Al_2_-O bond lengths decrease from 1.69 Å/1.45 Å to 1.55 Å/1.44 Å when the phase transition occurs at 1560 GPa, producing aluminum polyhedrons with coordination number of 8 rather than mixture of 7 and 8 in the U_2_S_3_-type phase.

Apart from Al_2_O_3_, we also identified aluminum oxides with uncommon stoichiometries, including Al_2_O, AlO, Al_4_O_7_, and AlO_3_. Static formation enthalpy calculations and phonon calculations provide evidence of the thermodynamically and dynamically stability of Al_2_O above 765 GPa, see in Fig. S2(a) and Fig. S5(a). It forms a *P4/nmm* phase over the whole pressure range that we investigated. Interestingly, the structure of the *P4/nmm* Al_2_O is similar to adding O atoms into the bcc phase of aluminum, in which the addition of O atoms makes the *c*-axis of the bcc lattice of Al atoms expend by 50% compared to the *a*-axis. As shown in Figure 1(d), the uneven distribution of O atoms forces some Al atoms to form square nets located on the ab plane. The AlO compound in Figure 1(e), becomes thermodynamically and dynamically stable and maintains a *P6_3_/mmc* structure above 1890 GPa, as shown in Fig. S2(b) and Fig. S6(a). The Al atoms form an hcp lattice rather than a bcc lattice, although in pure aluminum, the bcc phase is the most stable one at this pressure. The AlO_3_ compound becomes stable and maintains a $I{\bar{4}}_{3}d$ structure above 1260 GPa, see in Fig. S2(c) and Fig. S7(a). The $I{\bar{4}}_{3}d$ AlO_3_ in Figure 1(f) contains aluminum polyhedrons with a coordination number of 12, which is the largest known coordination number in the Al-O. Al_4_O_7_, shown in Fig. S1(b-d), forms a $P\bar{1}$ phase and a *Cmcm* phase from 850 GPa to 1500 GPa to 2000 GPa, respectively.

**Figure 2:**
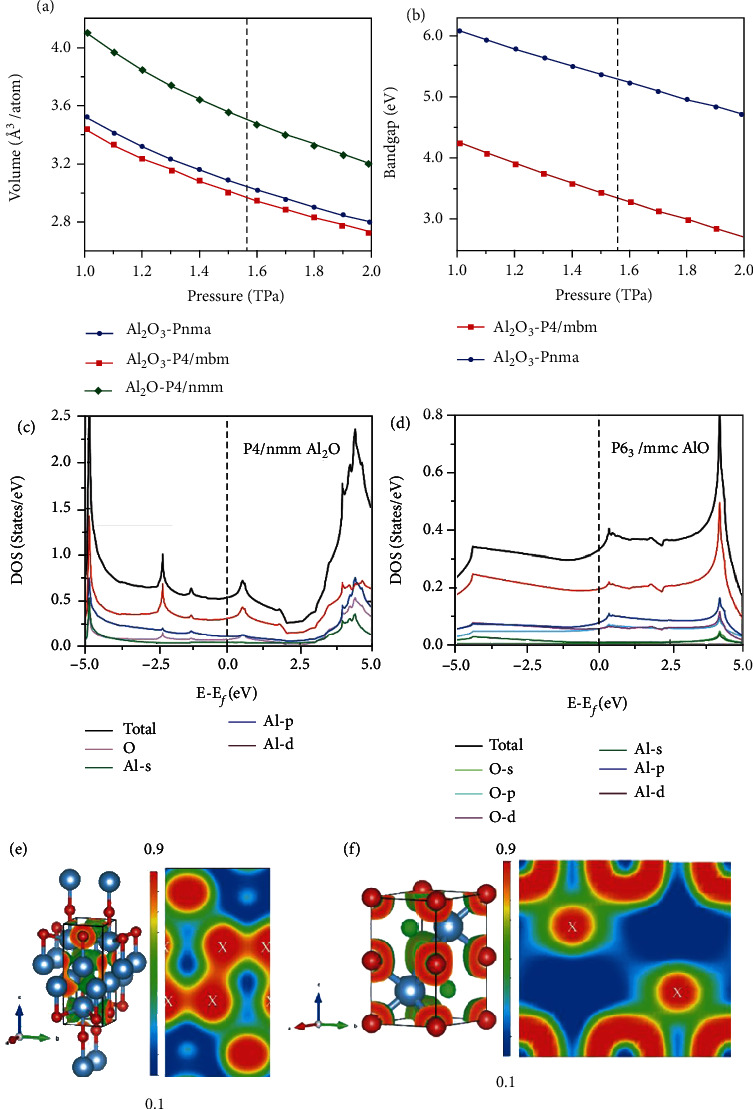
Equation of states (a) and electronic properties (b–f) of the newly found aluminum oxides, including bandgap (b), total and projected density of states (c, d), and electron localization functions (e, f). The white letters *X* represent the interstitial positions according to the results of Bader charge analysis. The vertical dashed lines in (a) and (b) represent the phase transition pressure from the U_2_S_3_-type alumina to the *P4/mbm* phase.

Furthermore, we investigated their physical properties, in particular, the equation of states and electronic properties. As shown in Figure 2, over the pressure range from 1 TPa to 2 TPa, the density of the *P4/mbm* alumina phase is just slightly higher than that of the U_2_S_3_-type alumina. However, in contrast to the U_2_S_3_-type alumina, the bandgap of the *P4/mbm* phase alumina is much smaller, decreasing from 5.21 eV to 3.28 eV at 1.6 TPa. As for Al_2_O, the results of density of state calculations suggest that it is metallic (see Figure 2(c)). The bands crossing the Fermi level (*E*_F_) are mainly composed of the d orbitals of Al atoms, and the conduction electrons occupying electronic states near *E*_F_ possess a connecting distribution between the two layers of Al atoms, as shown in Fig. S11. Also, the ELF in (110) plane displayed in Figure 2(e) shows that the *P4/nmm* Al_2_O, in analogy to high pressure electrides [43, 44], consists of ionic cores and localized electron density. According to the results of Bader charge analysis displayed in Table. S1, the Al atoms lose almost all the valence electrons, and the O atoms get about 1.7 electrons per atom. Other electrons (about 3.55 e) are localized in the interstitial space between the two layers of Al atoms, thus forming a connected electron localization region which coincides with the distribution of the conduction electrons. This suggests that the electron localization channels composed of the d orbitals of Al atoms contribute to the metallicity of the *P4/nmm* Al_2_O. In addition, the *P6_3_/mmc* AlO is metallic and shares electride features, which can be clearly observed in Figures 2(d) and 2(f). In contrast to *P4/nmm* Al_2_O, the electron localization region of the *P6_3_/mmc* AlO is isolated. Only about 0.03 electrons gather in the region centered on (0.667 0.333 0.75) and (0.333 0.667 0.25) to form pseudo anions.

**Figure 3:**
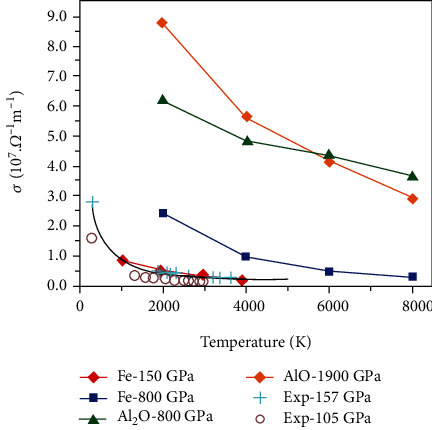
The electrical conductivity versus temperature of the *P4/nmm* Al_2_O at 800 GPa and *P6_3_/mmc* AlO at 1900 GPa, compared with that of the hcp iron. The simulation cells consist of 150, 162, and 144 atoms for iron, Al_2_O, and AlO, respectively. The cyan crosses are the experimental results by Ohta et al. [7] using LHDAC method, and the black line is fitted from their experimental data. The brown circles are the experimental results by Zhang et al. [45].

Since both the *P4/nmm* Al_2_O and the *P6_3_/mmc* AlO are metallic, we explored the values of their electrical conductivities and compared them with that of the hcp phase of iron [7, 45], which is the main component of Earth's core and has significant impact on Earth's dynamo. Several simulation methods have been employed to calculate the electrical conductivities of hcp iron under Earth's core conditions [6, 46, 47]. Here, we used the method of *Ab initial* molecular dynamics combined with the Kubo-Greenwood formula [40, 41]. We calculated the electrical conductivities of hcp iron at 150 GPa in the range of 1000-4000 K, together with both hcp iron and the *P4/nmm* Al_2_O at 800 GPa in the range of 2000-8000 K. Taking the influence of temperature on the crystal lattice into consideration, AIMD simulations were performed within the NVT ensemble. The effect of simulation cell size on the electrical conductivity has been tested by employing 128, 150, and 250 atoms for iron (see Fig. S12). For the hcp phase iron, our calculated electrical conductivity under 150 GPa is consistent with the experiment results under 157 GPa reported by Ohta et al. [7], confirming the feasibility of this calculation method. Moreover, with the increasing of temperature, the mean free path of electron will decrease down to the interatomic distance (the so-called Ioffe-Regel condition), resulting in resistivity saturation effects [48]. This is the reason that the electrical conductivities of both iron and Al_2_O converge to a constant at a higher temperature, as clearly shown in Figure 3. Most importantly, as shown in Table 1, we found that the electrical conductivity of Al_2_O under 800 GPa is much higher than that of iron in the temperature range of 2000-8000 K. The values of electrical conductivity of AlO under 1.9 TPa and those of Al_2_O under 800 GPa are in the same order of magnitude.

**Figure 4:**
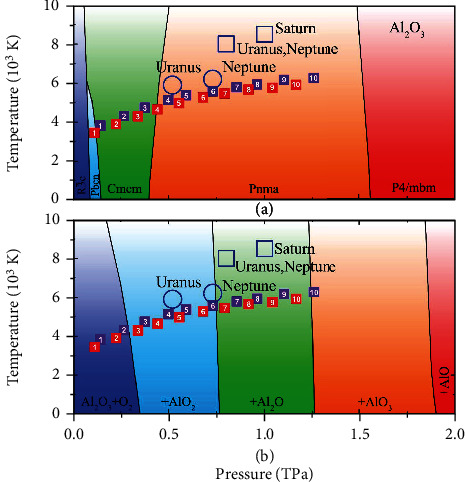
Proposed pressure-temperature phase diagrams of alumina (a) and other aluminum oxides (b). Phase boundary lines are marked with black solid lines. The violet and red squares display the pressure-temperature conditions at the core mantle boundary (CMB) of terrestrial and ocean type exoplanets, respectively, while numbers in those squares represent the planet mass in units of Earth mass (M_⊕_) [12]. Blue circles and squares mark out the estimated pressure-temperature conditions at CMB in the solar giant planets (Saturn, Uranus, and Neptune), according to the work by Guillot [11] and Nettelmann et al. [17], respectively.

With such high electrical conductivities, the aluminum suboxides could possibly influence the total conductivities of the planetary interior, which makes it necessary to explore the distribution of these newly found aluminum suboxides inside planets. Thus, we model the evolutions of these compounds at finite temperature conditions by quasiharmonic approximation. The calculated Gibbs free energy curves can help us to judge the most stable phase under finite temperature. We summarized our calculations up to 10,000 K in Figure 4, which provides an ultrahigh pressure-temperature phase diagram of alumina crystal and other aluminum oxides crystal. For Al_2_O_3_, the U_2_S_3_-type phase directly transforms into the *P4/mbm* phase at ~1560 GPa and shows less sensitivity with temperature. For other stoichiometries in the Al-O system, all of them show good thermal stability and will not decompose up to 10,000 K, which also agrees with our cross-checks with AIMD simulations.

Furthermore, the adiabatic geotherms (violet and red squares) suggested for super-Earths are also plotted in Figure 4 to illustrate the pressure-temperature conditions at the core-mantle boundary (CMB) with respect to different planet models [12]. When the planetary mass increases up to 4 M_⊕_, the U_2_S_3_-type alumina could gain stability inside both the terrestrial and ocean forming super-Earths. For other oxides, the *P2_1_/c* AlO_2_ could possibly exist in the core of terrestrial planets as well as ocean planets between ~3 M_⊕_ and ~6 M_⊕_, while the *P4/nmm* Al_2_O is expected to appear in those terrestrial planets weighing over 6 M_⊕_ and ocean planets weighing over 7 M_⊕_. For the planets in the solar system, the temperature and pressure conditions at their core mantle boundary (CMB) are not well-determined and are model dependent [11, 17]. The core mantle boundary of Neptune covers the stable temperature and pressure conditions of the *P4/nmm* Al_2_O, suggesting their possible appearance in Neptune's deep interior. In addition, the formation of the $I{\bar{4}}_{3}d$ AlO_3_, the *P4/mbm* alumina and the *P6_3_/mmc* AlO occur at much higher pressure and could exist in the deep interiors of Jupiter and Saturn (see in Fig. S13).

The above discussions about the stability of these unexpected aluminum oxides indicate the possible widespread relevance to the interior of planets. However, based on the dynamo theory [49, 50], it is reported that both convection of fluid and electrical conductivity are required. Under such extreme pressures, the aluminum suboxides cannot melt below 10000 K, which excludes convection and the possibility for generating the magnetic field by themselves. Still, the high electrical conductivity of Al_2_O may affect the total electrical conductivity of the planetary core and the magnetic fields indirectly. For instance, they can affect the evolution and distribution of conductive compounds in the interior of planets and contribute to the formation of the multidipole feature in the magnetic field of giant planets.

In conclusion, we explore the structures of Al-O system under extreme pressure extensively up to terapascal range and predict a ground state *P4/mbm* phase of Al_2_O_3_ and several compounds of aluminum suboxides and superoxides. The *P4/nmm* Al_2_O can survive in the core-mantle boundary of Uranus and Neptune, while the $I{\bar{4}}_{3}d$ AlO_3_, the *P4/mbm* Al_2_O_3_, and the *P6_3_/mmc* AlO might exist in the deep interiors of the outer planets in the solar system as well as super-Earth exoplanets. These predictions could be confirmed by shock-wave experiments [8, 42]. Furthermore, we find that the *P4/nmm* Al_2_O and the *P6_3_/mmc* AlO are metallic and have interesting features as electrides. At planetary core or mantle condition, the electrical conductivity of *P4/nmm* Al_2_O is about 4.34 × 10^7^ *Ω*^−1^ m^−1^, almost one order magnitude higher than that of iron at the same pressure-temperature condition, which could be important for understanding planetary conductivity.

## Figures and Tables

**Table 1:** The calculated electrical conductivities of hcp-Fe and the *P4/nmm* Al_2_O at 800 GPa and different temperatures.

Temperature (K)	*σ*_Fe_(*Ω*^−1^*m*^−1^)	*σ*_Al_2_O_(*Ω*^−1^*m*^−1^)	*σ*_Al_2_O_/*σ*_Fe_
2000	2.44 × 10^7^	6.18 × 10^7^	2.53
4000	9.63 × 10^6^	4.82 × 10^7^	5.01
6000	4.78 × 10^6^	4.34 × 10^7^	9.07
8000	2.92 × 10^6^	3.96 × 10^7^	12.65

## Data Availability

The data that support the plots within this paper and other findings of this study are available from the corresponding author upon reasonable request.

## Supplementary Materials

Supplementary Materials Figure S1: (a) enthalpies of the Al_2_O_3_ phases relative to the U_2_S_3_-type structure in the pressure range from 300 GPa to 2000 GPa. (b) Enthalpies of the Al_4_O_7_ phases relative to the Cmcm structure in the pressure range from 800 GPa to 2000 GPa. The crystal structures of the $\text{P}\overset{¯}{1}$ Al_4_O_7_ (c) and the Cmcm Al_4_O_7_ (d) at 900 and 1500 GPa, respectively. The red and silver spheres denote oxygen and aluminum atoms, respectively. Figure S2: convex hulls for the P4/nmm Al_2_O (a), the P6_3_/mmc AlO (b), the $\text{I}{\overset{¯}{4}}_{3}\text{d}$ AlO_3_ (c), the $\text{P}\overset{¯}{1}$ and Cmcm Al_4_O_7_ structures (d), respectively. Figure S3: convex hulls for the P4/nmm Al_2_O using different methods Figure S4: phonon dispersions and electronic band structures of the P4/mbm Al_2_O_3_ at 1600 GPa. Figure S5: phonon dispersions and electronic band structures of the P4/nmm Al_2_O at 800 GPa. Figure S6: phonon dispersions and electronic band structures of the P6_3_/mmc AlO at 2 TPa. Figure S7: phonon dispersions and electronic band structures of the $\text{I}{\overset{¯}{4}}_{3}\text{d}$ AlO_3_ at 1300 GPa. Figure S8: phonon dispersions and electronic band structures of the $\text{P}\overset{¯}{1}$ Al_4_O_7_ at 1000 GPa. Figure S9: phonon dispersions and electronic band structures of the Cmcm Al_4_O_7_. Figure S10: equation of states (a) and bandgap (b) of the newly found aluminum oxides. Figure S11: the projected band structure and the projection of the bands crossing the Fermi level for the P4/nmm Al_2_O (a, b) and the P6_3_/mmc AlO (c, d) in real space. Figure S12: the electrical conductivity versus temperature of the hcp iron at Earth's core conditions. Figure S13: proposed pressure-temperature phase diagrams of aluminum oxides up to 4 TPa and 16000 K. The $\text{I}{\overset{¯}{4}}_{3}\text{d}$ AlO_3_ becomes stable in area II. Figure S14: results of the variable-composition structure prediction at 2 TPa. Figure S15: The ELF of the $\text{I}{\overset{¯}{4}}_{3}\text{d}$ AlO_3_. The red spheres represent the O atoms, and the silver spheres represent the Al atoms. Table SI: Bader charges for atoms in the P4/nmm phase Al_2_O at 800 GPa. Table SII: Bader charges for atoms in the P6_3_/mmc phase AlO at 2 TPa. Table SIII: Bader charges for atoms in the P6_3_/mmc phase AlO_3_ at 1300 GPa. Crystal structure information (CIF file) for all the structures is found in this work.
