# Supplementary material for: Metallic Aluminum Suboxides with Ultrahigh Electrical Conductivity at High Pressure
Source: Research (Wash D C). 2022 Aug 28;2022:9798758. doi: 10.34133/2022/9798758 (PMC9448442; doi:10.34133/2022/9798758)
Supplement: Supplementary Materials — Figure S1: (a) enthalpies of the Al2O3 phases relative to the U2S3-type structure in the pressure range from 300 GPa to 2000 GPa. (b) Enthalpies of the Al4O7 phases relative to the Cmcm structure in the pressure range from 800 GPa to 2000 GPa. The crystal structures of the P1¯ Al4O7 (c) and the Cmcm Al4O7 (d) at 900 and 1500 GPa, respectively. The red and silver spheres denote oxygen and aluminum atoms, respectively. Figure S2: convex hulls for the P4/nmm Al2O (a), the P63/mmc AlO (b), the I4¯3d AlO3 (c), the P1¯ and Cmcm Al4O7 structures (d), respectively. Figure S3: convex hulls for the P4/nmm Al2O using different methods Figure S4: phonon dispersions and electronic band structures of the P4/mbm Al2O3 at 1600 GPa. Figure S5: phonon dispersions and electronic band structures of the P4/nmm Al2O at 800 GPa. Figure S6: phonon dispersions and electronic band structures of the P63/mmc AlO at 2 TPa. Figure S7: phonon dispersions and electronic band structures of the I4¯3d AlO3 at 1300 GPa. Figure S8: phonon dispersions and electronic band structures of the P1¯ Al4O7 at 1000 GPa. Figure S9: phonon dispersions and electronic band structures of the Cmcm Al4O7. Figure S10: equation of states (a) and bandgap (b) of the newly found aluminum oxides. Figure S11: the projected band structure and the projection of the bands crossing the Fermi level for the P4/nmm Al2O (a, b) and the P63/mmc AlO (c, d) in real space. Figure S12: the electrical conductivity versus temperature of the hcp iron at Earth's core conditions. Figure S13: proposed pressure-temperature phase diagrams of aluminum oxides up to 4 TPa and 16000 K. The I4¯3d AlO3 becomes stable in area II. Figure S14: results of the variable-composition structure prediction at 2 TPa. Figure S15: The ELF of the I4¯3d AlO3. The red spheres represent the O atoms, and the silver spheres represent the Al atoms. Table SI: Bader charges for atoms in the P4/nmm phase Al2O at 800 GPa. Table SII: Bader charges for atoms in the P63/mmc phas [file 9798758.f1.pdf]

Supplemental Information for "Metallic aluminum suboxides with ultra-high electrical conductivity at high pressure"

Tianheng Huang,[1] Cong Liu,[1] Junjie Wang,[1] Shuning Pan,[1] Yu Han,[1] Chris J. Pickard,[2,3] Ravit Helled,[4] Hui-Tian Wang,[1] Dingyu Xing,[1] and Jian Sun[1,*]

[1] *National Laboratory of Solid State Microstructures, School of Physics, and Collaborative Innovation Center of Advanced Microstructures, Nanjing University, Nanjing, 210093, China*

[2] *Department of Materials Science & Metallurgy, University of Cambridge, 27 Charles Babbage Road, Cambridge CB3 0FS, UK*

[3] *Advanced Institute for Materials Research, Tohoku University 2-1-1 Katahira, Aoba, Sendai, 980-8577, Japan*

[4] *Institute for Computational Science, Center for Theoretical Astrophysics & Cosmology, University of Zurich, Switzerland*

Supplemental Method

Details of the high-pressure crystal structure search

We performed fix-composition structure prediction for Al_2O_3 and variable-composition for Al-O system (from 1:6 to 6:1) at 200 GPa, 500 GPa, 750 GPa, 1 TPa, 1.5 TPa and 2 TPa. When searching for Al_2O_3, the maximum number of atoms in the unit cell is 30 and when performing variable-composition search, the number is 48. Extensive fix-compostion searches were performed for the stoichiometry where a new compound is found in the variable-composition search. In most of the searches, 900 crystal structures (15 generations, 60 structures per generation) were generated for us to select the most stable ones. To accelerate the searching, we added known aluminum oxides structures from previous work as part of initial seeds.

---

[*] Corresponding author. jiansun@nju.edu.cn

Details of the DFT calculations

We chose $3s^2 3p^1$ and $2s^2 2p^4$ as the valence electrons for Al and O while using the generalized gradient approximation (GGA) in the Perdew-Burke-Ernzerhof (PBE) exchange-correlation functional [1]. The plane-wave cutoff was set as 1050 eV and the Brillouin zone (BZ) was meshed choosing the gamma-centered Monkhorst-Pack approximately $2\pi \times 0.025$ Å^{-1}. When calculating the electrical conductivity of iron as a comparison, the valence electrons chosen for Fe were $4s^1 3d^7$ and single particle orbitals were expanded in plane waves with a cutoff of 420 eV. The Brillouin zone (BZ) was meshed choosing the gamma-centered Monkhorst-Pack approximately $2\pi \times 0.03$ Å^{-1} when calculating the transport properties. As for the *ab initio* molecular simulation part, the equations of motion were integrated with ionic time steps of 1 fs and the Brillouin zone was sampled at Γ point only. To ensure the volume of the cell remains reasonable under given pressure, we first run simulations with a *NPT* ensemble with 2 ps and take the average cell volume of the last 1000 configurations as the volume employed in the *NVT* simulations.

Due to the existence of the electron localization area in P4/nmm Al_2O and $P6_3$/mmc AlO, we need to adjust the Wigner-Seitz radius for each atoms when calculating the electron density of states [2], which is the parameter **RWIGS** as implemented in VASP code. Here we set **RWIGS** values in accordance with the results (atomic volumes) of the Bader charge calculations rather than the parameter written in the pseudopotential. The **RWIGS** for O atoms in P4/nmm Al_2O is 1.05 and the **RWIGS** for Al atoms is 1.2. As for the P63/mmc AlO, the **RWIGS** for O and Al atoms are 0.95 and 1.25, respectively.

To validate the dynamical stabilities of the predicted structures, we have investigated the phonon dispersion curves using $2 \times 2 \times 2$ supercells with PHONOPY code [3]. Also, we have investigated the anharmonic phonon dispersion of the Cmcm Al_4O_7 at 1500 GPa and 300 K using the DynaPhoPy code [4]. The trajectory was provided by the *Ab initio* molecular dynamics simulation with a $2 \times 2 \times 2$

supercell using VASP code.

Details of the formation energy cross-checks

We have performed cross-checks for the formation enthalpy of the Al_2O using WIEN2k, VASP and CASTEP software. In the structure searches with AIRSS, the CASTEP code was employed with similar convergence parameters as used in the Vienna *Ab initio* simulation package. For the calculations using WIEN2k code [5,6], the Perdew-Burke-Ernzerhof version of the generalized gradient approximation (GGA-PBE) was employed. For the calculations using VASP code, apart from the GGA-PBE methods, the hybrid exchange-correlation functional (HSE06) [7,8] was applied. For the calculations using CASTEP code [9], similar convergence parameters as used in VASP were employed.

Supplemental Figures

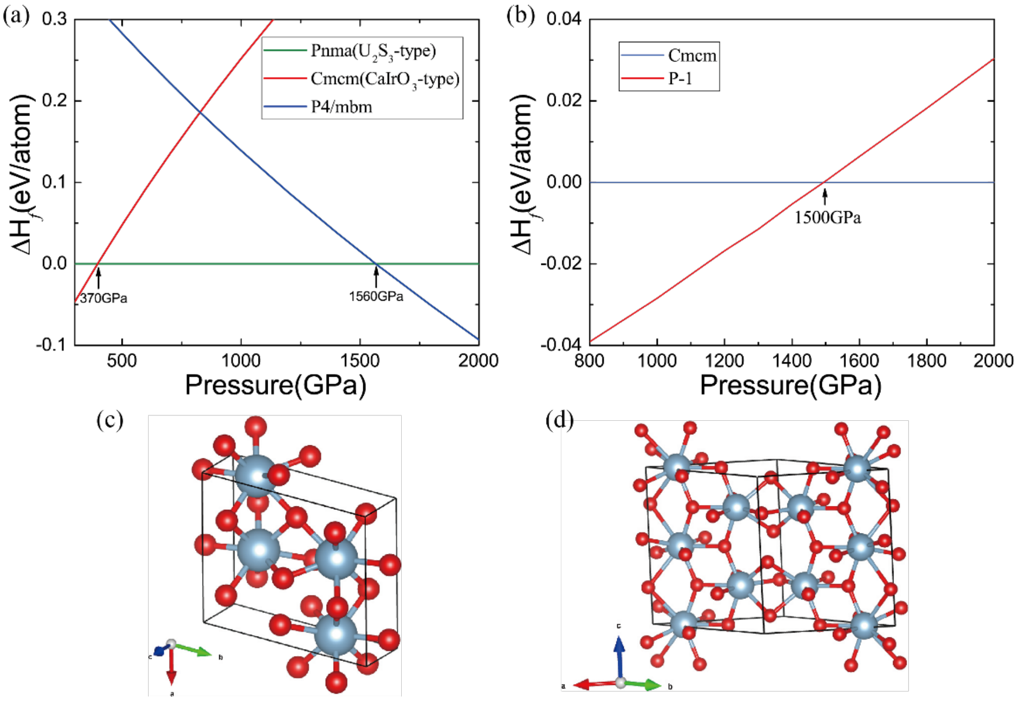

Fig. S1. (a) Enthalpies of the Al_2O_3 phases relative to the U_2S_3-type structure in the pressure range from 300 GPa to 2000 GPa. (b) Enthalpies of the Al_4O_7 phases relative to the Cmcm structure in the pressure range from 800 GPa to 2000 GPa. The crystal structures of the $P\bar{1}$ Al_4O_7 (c) and the Cmcm Al_4O_7 (d) at 900 and 1500 GPa, respectively. The red and silver spheres denote oxygen and aluminum atoms, respectively.

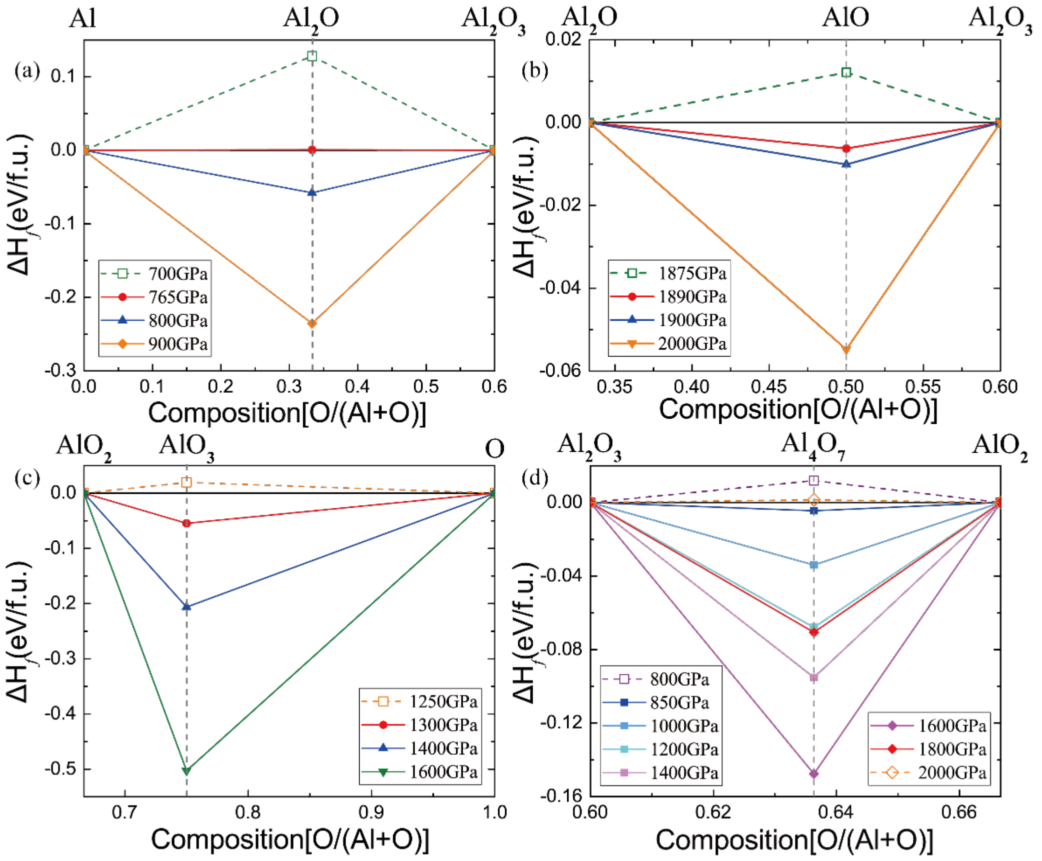

Fig. S2. Convex hulls for the P4/nmm Al_2O (a), the $P6_3/mmc$ AlO (b), the $I\bar{4}3d$ AlO_3 (c), the $P\bar{1}$ and Cmcm Al_4O_7 structures (d), respectively.

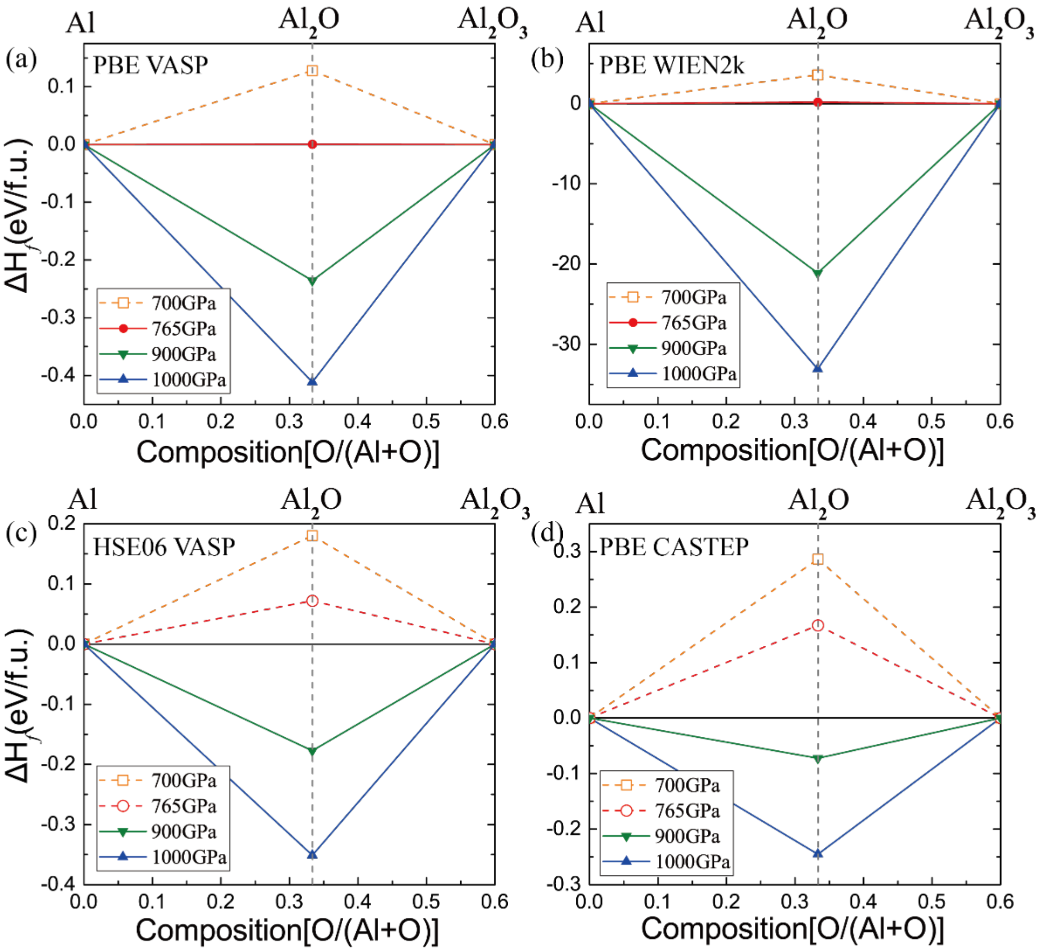

Fig. S3. Convex hulls for the P4/nmm Al_2O using different methods. (a) Perdew-Burke-Ernzerhof version of the generalized gradient approximation (GGA-PBE) was employed using VASP code. (b) Perdew-Burke-Ernzerhof version of the generalized gradient approximation (GGA-PBE) was employed using WIEN2k code. (c) Hybrid exchange-correlation functional (HSE06) was employed using VASP code. (d) Perdew-Burke-Ernzerhof functional was employed using CASTEP code.

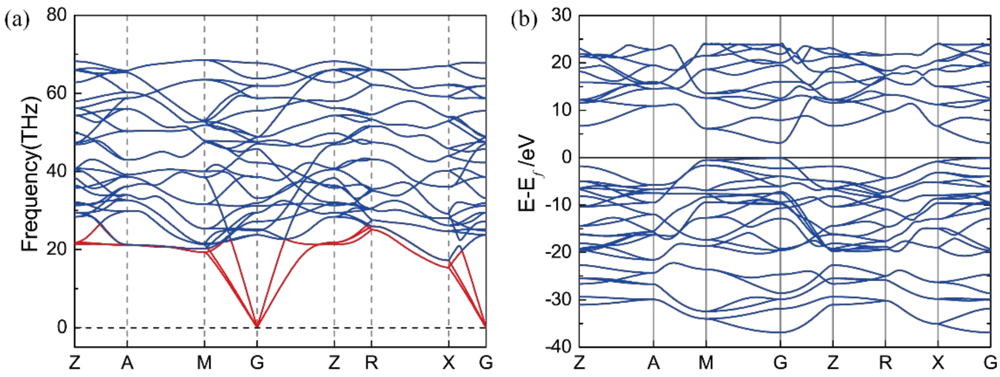

Fig. S4. Phonon dispersions and electronic band structures of the P4/mbm Al_2O_3 at 1600 GPa. Red and blue lines in Fig. (a) represent the acoustic and optical branches, respectively.

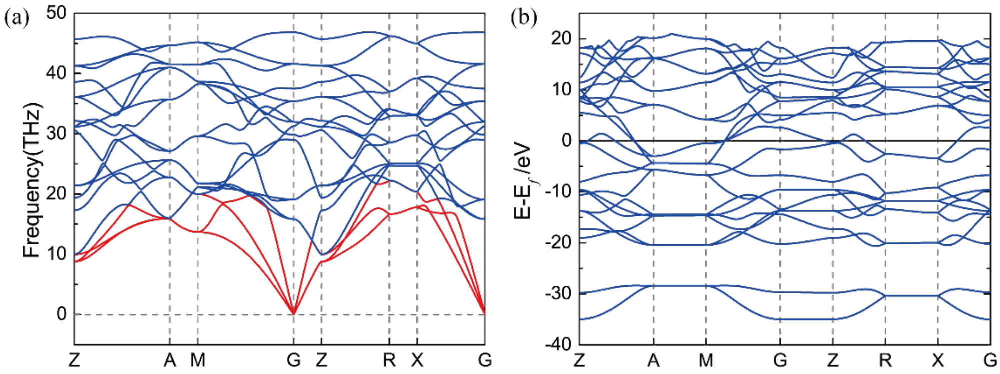

Fig. S5. Phonon dispersions and electronic band structures of the P4/nmm Al_2O at 800 GPa. Red and blue lines in Fig. (a) represent the acoustic and optical branches, respectively.

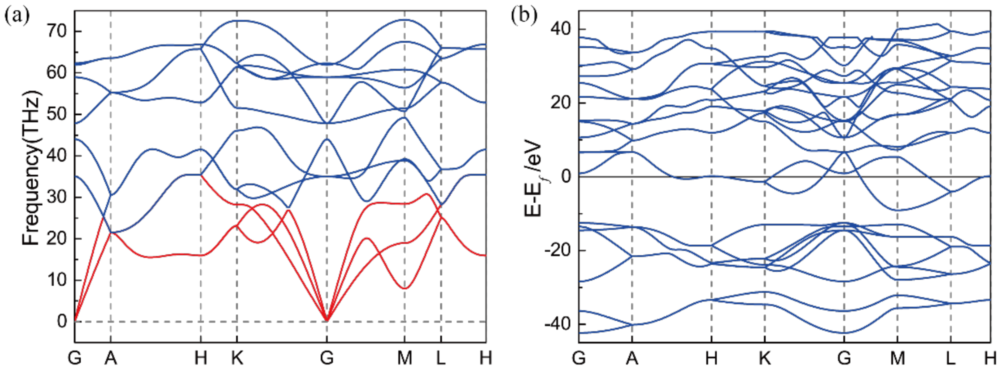

Fig. S6. Phonon dispersions and electronic band structures of the P6$_3$/mmc AlO at 2 TPa. Red and blue lines in Fig. (a) represent the acoustic and optical branches, respectively.

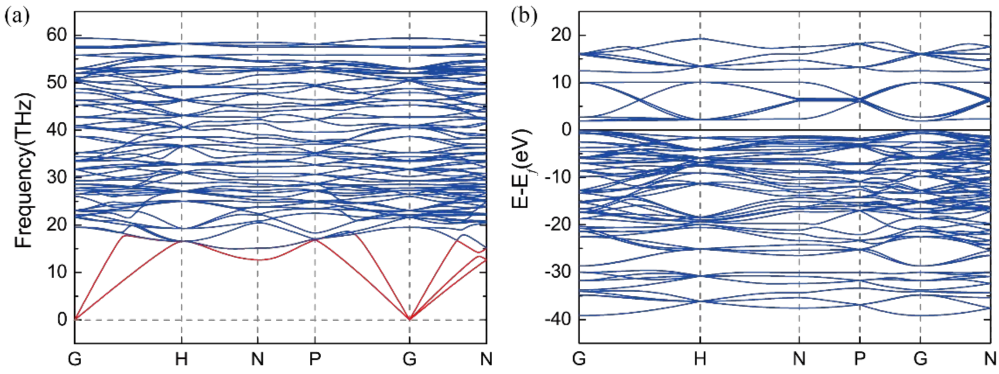

Fig. S7. Phonon dispersions and electronic band structures of the I$\bar{4}$3d AlO_3 at 1300 GPa. Red and blue lines in Fig. (a) represent the acoustic and optical branches, respectively.

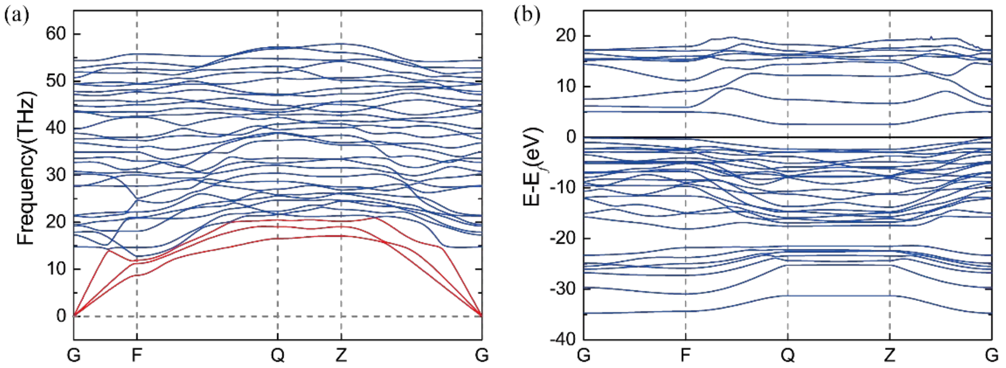

Fig. S8. Phonon dispersions and electronic band structures of the $P\bar{1}$ Al_4O_7 at 1000 GPa. Red and blue lines in Fig. (a) represent the acoustic and optical branches, respectively.

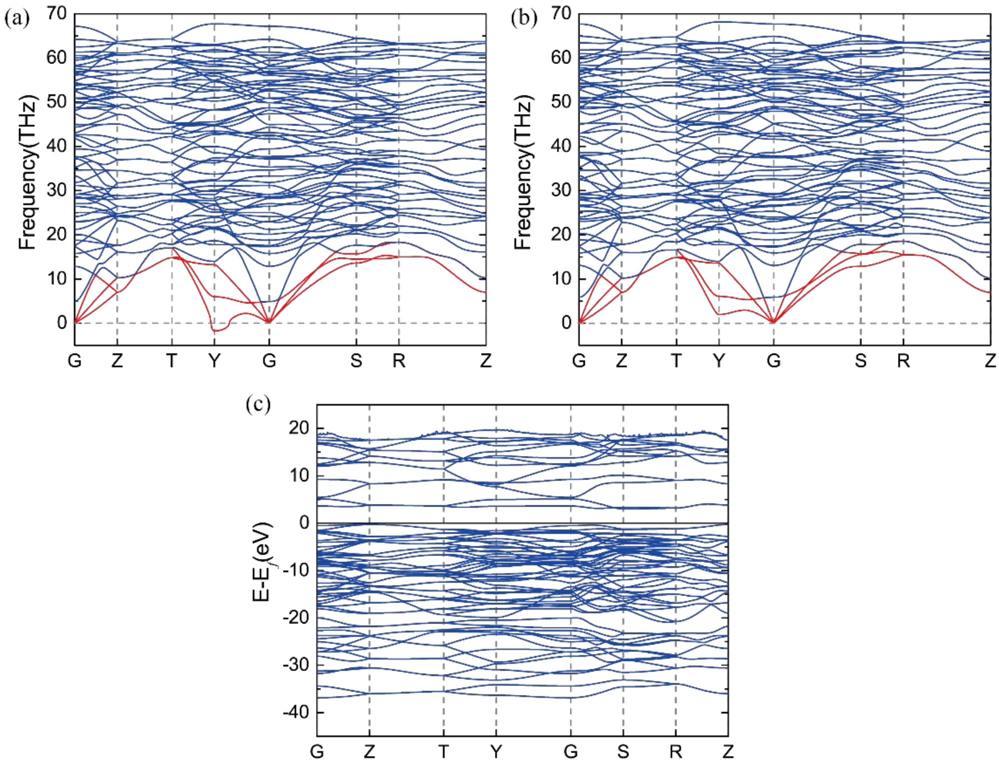

Fig. S9. Phonon dispersions and electronic band structures of the Cmcm Al_4O_7. (a) Phonon dispersions at 1500 GPa. (b) Phonon dispersions with anharmonic effect at 300 K. (c) Electronic band structures at 1500 GPa. Red and blue lines in phonon dispersions represent the acoustic and optical branches, respectively.

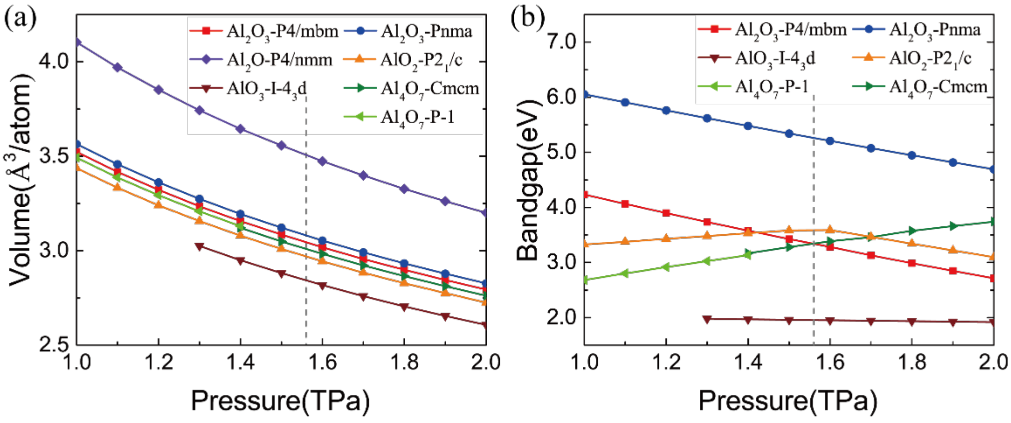

Fig. S10. Equation of states (a) and bandgap (b) of the newly found aluminum oxides.

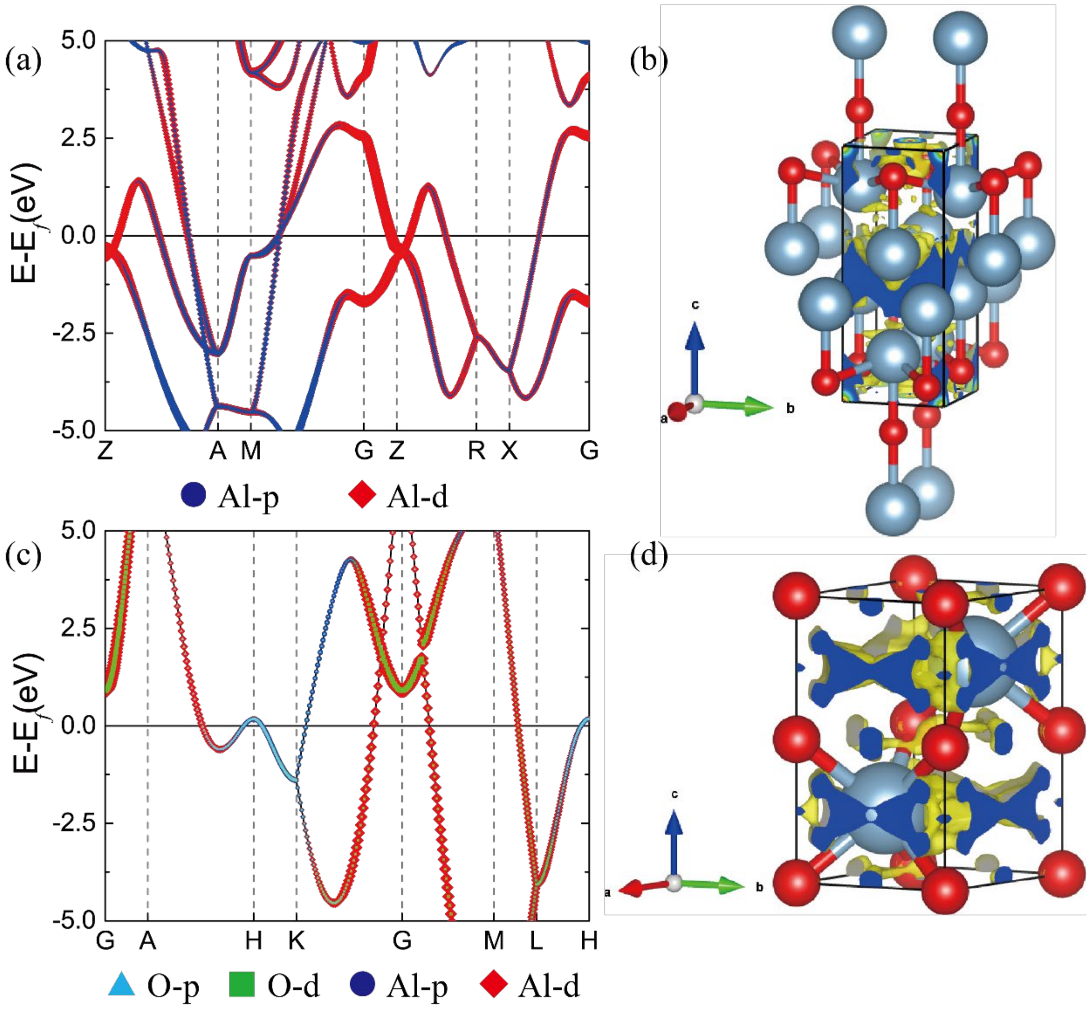

Fig. S11. The projected band structure and the projection of the bands crossing the Fermi level for the P4/nmm Al_2O (a-b) and the $P6_3/mmc$ AlO (c-d) in real space. The cyan triangles, green squares, blue circles and red diamonds in Fig. (a) and (c) represent the contribution of the p and d orbitals of O and Al atoms, respectively. The energy interval chosen in Fig. (b) and (d) is from -0.5 eV to 0.5 eV.

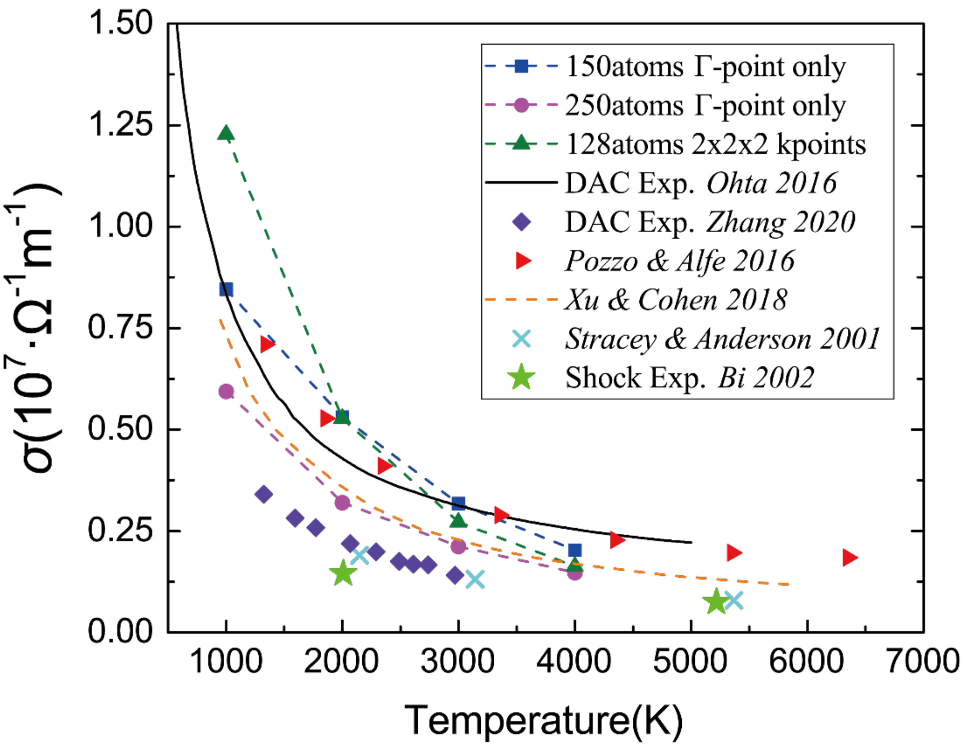

Fig. S12. The electrical conductivity versus temperature of the hcp iron at Earth's core conditions. Results were obtained using 128, 150 and 250 atoms system, using 2×2×2 k-points and Gamma point only. The black line is fitted from the experimental results by Ohta *et al*. [10]. The purple diamonds are the experimental results by Zhang *et al.* [11]. The red triangles are AIMD results from Pozzo and Alfe's work in 2016 [12]. The orange dash line is DFPT results taking saturation effects into consideration according to Cohen's data in 2018 [13]. The crosses are extrapolations to this density using the systematics of Stacey and Anderson based on the melting curve [14]. The green stars are experimental results by Bi *et al.* using shock-wave compression [15].

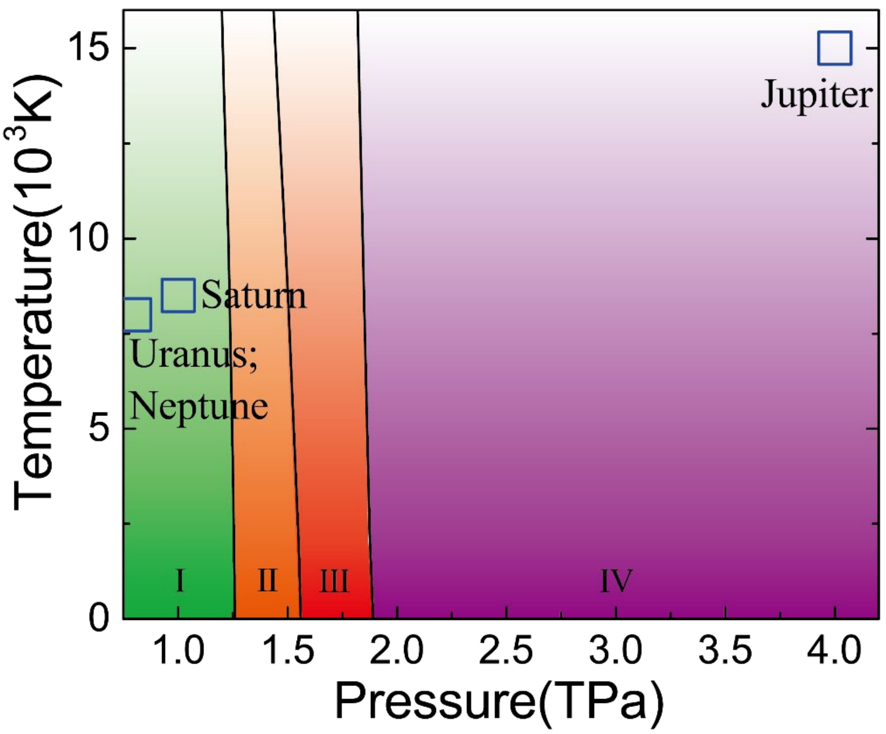

Fig. S13. Proposed pressure-temperature phase diagrams of aluminum oxides up to 4TPa and 16000K. The $I\bar{4}3d$ AlO_3 becomes stable in area II. The U_2S_3-type Al_2O_3 remain stable in area I and II, while the P4/mbm Al_2O_3 become stable in area III. Both the P4/mbm Al_2O_3 and the $P6_3/mmc$ AlO survive in area IV. Blue squares mark out the estimated pressure-temperature conditions at CMB in the solar giant planets (Uranus, Neptune, Saturn and Jupiter), according to the work by Guillot *et al*. [16].

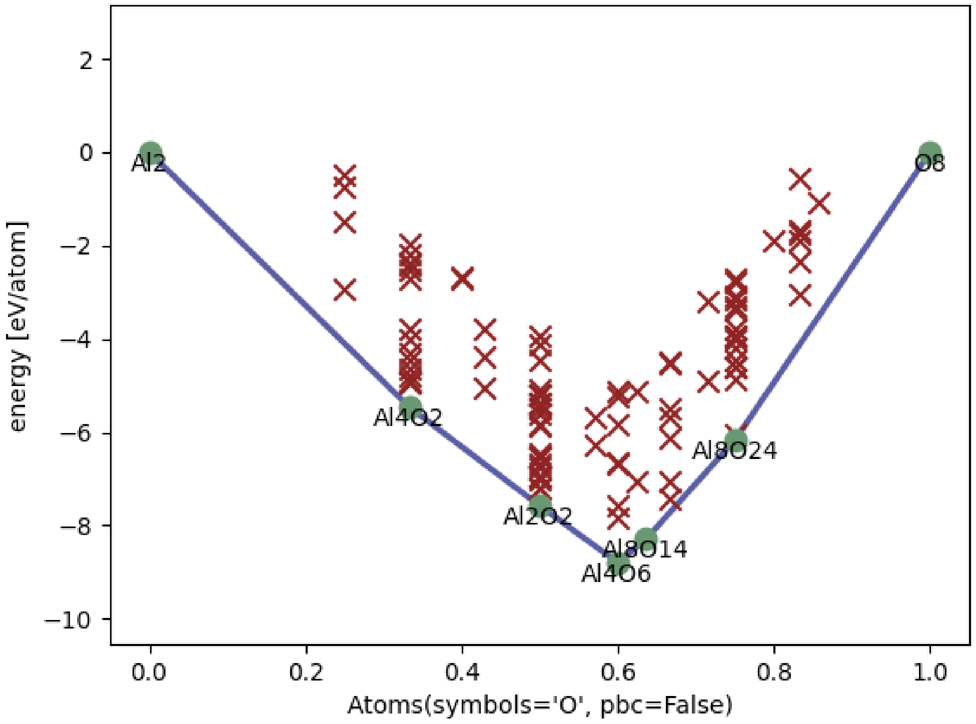

Fig. S14. Results of the variable-compositions strcutrue prediction at 2 TPa. The blue line represents the convex hell. The green points represent the thermodynamically stable structures and the red crosses represent the thermodynamically unstable structures.

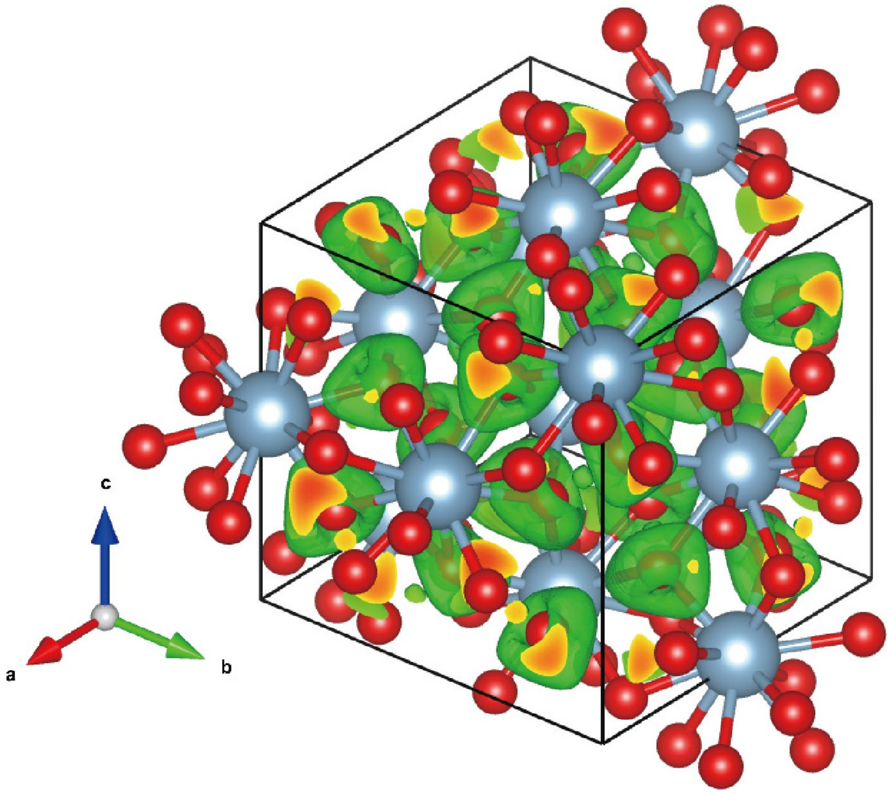

Fig. S15. The ELF of the I-4$_3$d AlO_3. The red spheres represent the O atoms and the sliver spheres represent the Al atoms. The green area around the O atoms is the electron localization region.

Table. SI. Bader charges for atoms in the P4/nmm phase Al_2O at 800 GPa.

#	X	Y	Z	CHARGE(e)
O	0.00000	0.50000	0.89054	7.722965
O	0.50000	0.00000	0.10839	7.733688
Al	0.50000	0.00000	0.81633	0.718585
Al	0.00000	0.50000	0.18343	0.718585
isp(X)	0.00000	0.50000	0.59184	0.884515
isp(X)	0.50000	0.00000	0.37106	1.340238
isp(X)	0.00000	0.00000	0.40816	0.884643
isp(X)	0.50000	0.50000	0.62523	1.328445
isp(X)	0.00000	0.00000	0.37106	1.339419
isp(X)	0.00000	0.00000	0.62523	1.329239

NUMBER OF ELECTRONS: 24.0000

Table. SII. Bader charges for atoms in the $P6_3/mmc$ phase AlO at 2 TPa.

#	X	Y	Z	CHARGE(e)
O	0.00000	0.00000	0.50000	7.887712
O	0.00000	0.00000	0.00000	7.887714
Al	0.66667	0.33333	0.25000	0.814927
Al	0.33333	0.66667	0.75000	0.814869
isp(X)	0.33333	0.66667	0.25000	0.027933
isp(X)	0.66667	0.33333	0.75000	0.027914

NUMBER OF ELECTRONS: 18.0000

Table. SIII. Bader charges for atoms in the $P6_3/mmc$ phase AlO3 at 1300 GPa.

#	X	Y	Z	CHARGE(e)
O	0.035259	0.830392	4.308448	6.800009
O	2.857206	-0.830392	1.415983	6.788354
O	2.857206	0.830392	1.476482	6.792030
O	2.927724	2.062073	1.476482	6.796171
O	4.308448	0.035259	0.830392	6.800009
O	1.415983	2.857206	-0.830392	6.788354
O	1.476482	2.857206	0.830392	6.792030
O	1.476482	2.927724	2.062073	6.793651
O	0.830392	4.308448	0.035259	6.800009
O	-0.830392	1.415983	2.857206	6.788354
O	0.830392	1.476482	2.857206	6.792030
O	2.062073	1.476482	2.927724	6.793651
O	2.276624	1.481492	-0.030249	6.800009
O	3.508306	1.410973	-0.030249	6.788354
O	2.276624	-1.481492	2.922714	6.792030
O	0.615841	1.481492	0.030249	6.793651
O	1.481492	-0.030249	2.276624	6.800009
O	1.410973	-0.030249	3.508306	6.788354
O	-1.481492	2.922714	2.276624	6.792030
O	1.481492	0.030249	0.615841	6.796171
O	-0.030249	2.276624	1.481492	6.800009
O	-0.030249	3.508306	1.410973	6.788354
O	2.922714	2.276624	-1.481492	6.792030
O	0.030249	0.615841	1.481492	6.793651
Al	1.393538	-1.393538	1.498927	0.618659
Al	1.498927	1.393538	-1.393538	0.618659
Al	-1.393538	1.498927	1.393538	0.618659

Al	1.393538	1.393538	1.393538	0.619372
Al	2.945160	-0.052695	0.052695	0.618659
Al	-0.052695	0.052695	2.945160	0.618659
Al	0.052695	2.945160	-0.052695	0.618659
Al	2.839770	2.839770	2.839770	0.619372

NUMBER OF ELECTRONS: 168.0000

Crystal structure information (CIF file) for all the structures found in this work

data_Al2O3-P4mbm-1600GPa
_audit_creation_date 2021-08-13
_audit_creation_method 'Materials Studio'
_symmetry_space_group_name_H-M 'P4/MBM'
_symmetry_Int_Tables_number 127
_symmetry_cell_setting tetragonal
loop_
_symmetry_equiv_pos_as_xyz
  x,y,z
  -x,-y,z
  -y,x,z
  y,-x,z
  -x+1/2,y+1/2,-z
  x+1/2,-y+1/2,-z
  y+1/2,x+1/2,-z
  -y+1/2,-x+1/2,-z
  -x,-y,-z
  x,y,-z
  y,-x,-z
  -y,x,-z
  x+1/2,-y+1/2,z
  -x+1/2,y+1/2,z
  -y+1/2,-x+1/2,z
  y+1/2,x+1/2,z
_cell_length_a 3.9697
_cell_length_b 3.9697
_cell_length_c 1.9151
_cell_angle_alpha 90.0000
_cell_angle_beta 90.0000
_cell_angle_gamma 90.0000
loop_
_atom_site_label
_atom_site_type_symbol
_atom_site_fract_x
_atom_site_fract_y
_atom_site_fract_z
_atom_site_U_iso_or_equiv
_atom_site_adp_type
_atom_site_occupancy

O1	O	0.00000	0.00000	0.00000	0.01267	Uiso	1.00
O3	O	0.14398	0.35602	0.50000	0.01267	Uiso	1.00
Al1	Al	0.33887	0.16113	-0.00000	0.01267	Uiso	1.00

```
data_Al2O-P4nmm-800GPa
_audit_creation_date              2021-08-13
_audit_creation_method            'Materials Studio'
_symmetry_space_group_name_H-M    'P4/NMM'
_symmetry_Int_Tables_number       129
_symmetry_cell_setting            tetragonal
loop_
_symmetry_equiv_pos_as_xyz
  x,y,z
  -x,-y,z
  -y+1/2,x+1/2,z
  y+1/2,-x+1/2,z
  -x+1/2,y+1/2,-z
  x+1/2,-y+1/2,-z
  y,x,-z
  -y,-x,-z
  -x+1/2,-y+1/2,-z
  x+1/2,y+1/2,-z
  y,-x,-z
  -y,x,-z
  x,-y,z
  -x,y,z
  -y+1/2,-x+1/2,z
  y+1/2,x+1/2,z
_cell_length_a                    2.2200
_cell_length_b                    2.2200
_cell_length_c                    5.3926
_cell_angle_alpha                 90.0000
_cell_angle_beta                  90.0000
_cell_angle_gamma                 90.0000
loop_
_atom_site_label
_atom_site_type_symbol
_atom_site_fract_x
_atom_site_fract_y
_atom_site_fract_z
_atom_site_U_iso_or_equiv
_atom_site_adp_type
_atom_site_occupancy
O1     O     0.00000   0.50000   0.89104   0.01267  Uiso   1.00
Al1    Al    0.50000   0.00000   0.41199   0.01267  Uiso   1.00
Al3    Al    0.50000   0.00000   0.81340   0.01267  Uiso   1.00
```

```
data_AlO-P63mmc-2000GPa
_audit_creation_date              2021-08-13
_audit_creation_method            'Materials Studio'
_symmetry_space_group_name_H-M    'P63/MMC'
_symmetry_Int_Tables_number       194
_symmetry_cell_setting            hexagonal
loop_
_symmetry_equiv_pos_as_xyz
  x,y,z
  -y,x-y,z
  -x+y,-x,z
  -x,-y,z+1/2
  y,-x+y,z+1/2
  x-y,x,z+1/2
  y,x,-z
  x-y,-y,-z
  -x,-x+y,-z
  -y,-x,-z+1/2
  -x+y,y,-z+1/2
  x,x-y,-z+1/2
  -x,-y,-z
  y,-x+y,-z
  x-y,x,-z
  x,y,-z+1/2
  -y,x-y,-z+1/2
  -x+y,-x,-z+1/2
  -y,-x,z
  -x+y,y,z
  x,x-y,z
  y,x,z+1/2
  x-y,-y,z+1/2
  -x,-x+y,z+1/2
_cell_length_a                    1.9334
_cell_length_b                    1.9334
_cell_length_c                    3.5986
_cell_angle_alpha                 90.0000
_cell_angle_beta                  90.0000
_cell_angle_gamma                 120.0000
loop_
_atom_site_label
_atom_site_type_symbol
_atom_site_fract_x
_atom_site_fract_y
_atom_site_fract_z
```

_atom_site_U_iso_or_equiv
_atom_site_adp_type
_atom_site_occupancy
O1 O 0.00000 0.00000 0.50000 0.01267 Uiso 1.00
Al1 Al 0.66667 0.33333 0.25000 0.01267 Uiso 1.00

```
data_AlO3-I-43d-1300GPa
_audit_creation_date              2022-01-11
_audit_creation_method            'Materials Studio'
_symmetry_space_group_name_H-M    ' '
_symmetry_Int_Tables_number       0
_symmetry_cell_setting            cubic
loop_
_symmetry_equiv_pos_as_xyz
  x,y,z
  y-z+1/2,x-z,-z+1/2
  -y+z,-y+1/2,x-y+1/2
  -x+1/2,-x+z+1/2,-x+y
  z,x,y
  -z+1/2,y-z+1/2,x-z
  x-y+1/2,-y+z,-y+1/2
  -x+y,-x+1/2,-x+z+1/2
  y,z,x
  x-z,-z+1/2,y-z+1/2
  -y+1/2,x-y+1/2,-y+z
  -x+z+1/2,-x+y,-x+1/2
  y+1/2,x+1/2,z+1/2
  x-z+1/2,y-z,-z
  -y,-y+z+1/2,x-y
  -x+z,-x,-x+y+1/2
  x+1/2,z+1/2,y+1/2
  y-z,-z,x-z+1/2
  -y+z+1/2,x-y,-y
  -x,-x+y+1/2,-x+z
  z+1/2,y+1/2,x+1/2
  -z,x-z+1/2,y-z
  x-y,-y,-y+z+1/2
  -x+y+1/2,-x+z,-x
_cell_length_a                    5.0099
_cell_length_b                    5.0099
_cell_length_c                    5.0099
_cell_angle_alpha                 109.4712
_cell_angle_beta                  109.4712
_cell_angle_gamma                 109.4712
loop_
_atom_site_label
_atom_site_type_symbol
_atom_site_fract_x
_atom_site_fract_y
_atom_site_fract_z
```

_atom_site_U_iso_or_equiv
_atom_site_adp_type
_atom_site_occupancy
O1 O 1.10123 -0.13745 0.75087 0.01267 Uiso 1.00
Al1 Al 1.00000 0.48178 0.50000 0.01267 Uiso 1.00

data_Al4O7-P-1-900GPa
_audit_creation_date 2022-01-11
_audit_creation_method 'Materials Studio'
_symmetry_space_group_name_H-M 'P-1'
_symmetry_Int_Tables_number 2
_symmetry_cell_setting triclinic
loop_
_symmetry_equiv_pos_as_xyz
x,y,z
-x,-y,-z
_cell_length_a 4.2759
_cell_length_b 4.7190
_cell_length_c 2.0713
_cell_angle_alpha 102.4138
_cell_angle_beta 89.2233
_cell_angle_gamma 77.1252
loop_
_atom_site_label
_atom_site_type_symbol
_atom_site_fract_x
_atom_site_fract_y
_atom_site_fract_z
_atom_site_U_iso_or_equiv
_atom_site_adp_type
_atom_site_occupancy

O1	O	-0.09132	0.86624	0.18349	0.01267	Uiso	1.00
O3	O	0.59490	0.12246	0.81071	0.01267	Uiso	1.00
O4	O	0.34961	0.50087	0.94728	0.01267	Uiso	1.00
Al1	Al	0.68683	0.76331	0.64425	0.01267	Uiso	1.00
Al3	Al	0.17981	0.76766	0.62348	0.01267	Uiso	1.00
O2	O	0.00000	0.50000	0.50000	0.01267	Uiso	1.00

```
data_Al4O7-Cmcm-1500GPa
_audit_creation_date              2022-01-11
_audit_creation_method            'Materials Studio'
_symmetry_space_group_name_H-M    ' '
_symmetry_Int_Tables_number       0
_symmetry_cell_setting            orthorhombic
loop_
_symmetry_equiv_pos_as_xyz
  x,y,z
  -x,-y,z+1/2
  y,x,-z+1/2
  -y,-x,-z
  -x,-y,-z
  x,y,-z+1/2
  -y,-x,z+1/2
  y,x,z
_cell_length_a                    4.5191
_cell_length_b                    4.5191
_cell_length_c                    5.5073
_cell_angle_alpha                 90.0000
_cell_angle_beta                  90.0000
_cell_angle_gamma                 143.3826
loop_
_atom_site_label
_atom_site_type_symbol
_atom_site_fract_x
_atom_site_fract_y
_atom_site_fract_z
_atom_site_U_iso_or_equiv
_atom_site_adp_type
_atom_site_occupancy
O1     O    -0.31044  -0.68956   0.50000   0.01267  Uiso   1.00
Al5    Al   -0.87209  -0.12791   0.50000   0.01267  Uiso   1.00
O5     O    -0.78732  -0.16302   1.25000   0.01267  Uiso   1.00
Al1    Al   -0.35239  -0.61776   1.25000   0.01267  Uiso   1.00
O9     O    -0.67192  -0.67192   1.07906   0.01267  Uiso   1.00
O13    O    -0.85737  -0.85737   0.75000   0.01267  Uiso   1.00
```

```
data_Al-bcc
_audit_creation_date              2020-10-07
_audit_creation_method            'Materials Studio'
_symmetry_space_group_name_H-M    'IM-3M'
_symmetry_Int_Tables_number       229
_symmetry_cell_setting            cubic
loop_
_symmetry_equiv_pos_as_xyz
  x,y,z
  -x,-y,z
  -x,y,-z
  x,-y,-z
  z,x,y
  z,-x,-y
  -z,-x,y
  -z,x,-y
  y,z,x
  -y,z,-x
  y,-z,-x
  -y,-z,x
  y,x,-z
  -y,-x,-z
  y,-x,z
  -y,x,z
  x,z,-y
  -x,z,y
  -x,-z,-y
  x,-z,y
  z,y,-x
  z,-y,x
  -z,y,x
  -z,-y,-x
  -x,-y,-z
  x,y,-z
  x,-y,z
  -x,y,z
  -z,-x,-y
  -z,x,y
  z,x,-y
  z,-x,y
  -y,-z,-x
  y,-z,x
  -y,z,x
  y,z,-x
```

-y,-x,z
y,x,z
-y,x,-z
y,-x,-z
-x,-z,y
x,-z,-y
x,z,y
-x,z,-y
-z,-y,x
-z,y,-x
z,-y,-x
z,y,x
x+1/2,y+1/2,z+1/2
-x+1/2,-y+1/2,z+1/2
-x+1/2,y+1/2,-z+1/2
x+1/2,-y+1/2,-z+1/2
z+1/2,x+1/2,y+1/2
z+1/2,-x+1/2,-y+1/2
-z+1/2,-x+1/2,y+1/2
-z+1/2,x+1/2,-y+1/2
y+1/2,z+1/2,x+1/2
-y+1/2,z+1/2,-x+1/2
y+1/2,-z+1/2,-x+1/2
-y+1/2,-z+1/2,x+1/2
y+1/2,x+1/2,-z+1/2
-y+1/2,-x+1/2,-z+1/2
y+1/2,-x+1/2,z+1/2
-y+1/2,x+1/2,z+1/2
x+1/2,z+1/2,-y+1/2
-x+1/2,z+1/2,y+1/2
-x+1/2,-z+1/2,-y+1/2
x+1/2,-z+1/2,y+1/2
z+1/2,y+1/2,-x+1/2
z+1/2,-y+1/2,x+1/2
-z+1/2,y+1/2,x+1/2
-z+1/2,-y+1/2,-x+1/2
-x+1/2,-y+1/2,-z+1/2
x+1/2,y+1/2,-z+1/2
x+1/2,-y+1/2,z+1/2
-x+1/2,y+1/2,z+1/2
-z+1/2,-x+1/2,-y+1/2
-z+1/2,x+1/2,y+1/2
z+1/2,x+1/2,-y+1/2
z+1/2,-x+1/2,y+1/2

```
   -y+1/2,-z+1/2,-x+1/2
   y+1/2,-z+1/2,x+1/2
   -y+1/2,z+1/2,x+1/2
   y+1/2,z+1/2,-x+1/2
   -y+1/2,-x+1/2,z+1/2
   y+1/2,x+1/2,z+1/2
   -y+1/2,x+1/2,-z+1/2
   y+1/2,-x+1/2,-z+1/2
   -x+1/2,-z+1/2,y+1/2
   x+1/2,-z+1/2,-y+1/2
   x+1/2,z+1/2,y+1/2
   -x+1/2,z+1/2,-y+1/2
   -z+1/2,-y+1/2,x+1/2
   -z+1/2,y+1/2,-x+1/2
   z+1/2,-y+1/2,-x+1/2
   z+1/2,y+1/2,x+1/2
_cell_length_a                          3.2205
_cell_length_b                          3.2205
_cell_length_c                          3.2205
_cell_angle_alpha                       90.0000
_cell_angle_beta                        90.0000
_cell_angle_gamma                       90.0000
loop_
_atom_site_label
_atom_site_type_symbol
_atom_site_fract_x
_atom_site_fract_y
_atom_site_fract_z
_atom_site_U_iso_or_equiv
_atom_site_adp_type
_atom_site_occupancy
Al0    Al    0.00000    0.00000    0.00000    0.00000   Uiso    1.00
```

```
data_O2-R-3m
_audit_creation_date              2020-10-24
_audit_creation_method            'Materials Studio'
_symmetry_space_group_name_H-M    'R-3M'
_symmetry_Int_Tables_number       166
_symmetry_cell_setting            trigonal
loop_
_symmetry_equiv_pos_as_xyz
  x,y,z
  -y,x-y,z
  -x+y,-x,z
  y,x,-z
  x-y,-y,-z
  -x,-x+y,-z
  -x,-y,-z
  y,-x+y,-z
  x-y,x,-z
  -y,-x,z
  -x+y,y,z
  x,x-y,z
  x+2/3,y+1/3,z+1/3
  -y+2/3,x-y+1/3,z+1/3
  -x+y+2/3,-x+1/3,z+1/3
  y+2/3,x+1/3,-z+1/3
  x-y+2/3,-y+1/3,-z+1/3
  -x+2/3,-x+y+1/3,-z+1/3
  -x+2/3,-y+1/3,-z+1/3
  y+2/3,-x+y+1/3,-z+1/3
  x-y+2/3,x+1/3,-z+1/3
  -y+2/3,-x+1/3,z+1/3
  -x+y+2/3,y+1/3,z+1/3
  x+2/3,x-y+1/3,z+1/3
  x+1/3,y+2/3,z+2/3
  -y+1/3,x-y+2/3,z+2/3
  -x+y+1/3,-x+2/3,z+2/3
  y+1/3,x+2/3,-z+2/3
  x-y+1/3,-y+2/3,-z+2/3
  -x+1/3,-x+y+2/3,-z+2/3
  -x+1/3,-y+2/3,-z+2/3
  y+1/3,-x+y+2/3,-z+2/3
  x-y+1/3,x+2/3,-z+2/3
  -y+1/3,-x+2/3,z+2/3
  -x+y+1/3,y+2/3,z+2/3
  x+1/3,x-y+2/3,z+2/3
```

```
_cell_length_a                  1.7023
_cell_length_b                  1.7023
_cell_length_c                  7.4994
_cell_angle_alpha               90.0000
_cell_angle_beta                90.0000
_cell_angle_gamma               120.0000
loop_
_atom_site_label
_atom_site_type_symbol
_atom_site_fract_x
_atom_site_fract_y
_atom_site_fract_z
_atom_site_U_iso_or_equiv
_atom_site_adp_type
_atom_site_occupancy
O     O     1.33333   0.66667   -0.26134   0.00000  Uiso   1.00
loop_
_geom_bond_atom_site_label_1
_geom_bond_atom_site_label_2
_geom_bond_distance
_geom_bond_site_symmetry_2
_ccdc_geom_bond_type
O     O      1.080   16_544 S
```

```
data_O2-R-3m
_audit_creation_date              2020-10-24
_audit_creation_method            'Materials Studio'
_symmetry_space_group_name_H-M    'R-3M'
_symmetry_Int_Tables_number       166
_symmetry_cell_setting            trigonal
loop_
_symmetry_equiv_pos_as_xyz
  x,y,z
  -y,x-y,z
  -x+y,-x,z
  y,x,-z
  x-y,-y,-z
  -x,-x+y,-z
  -x,-y,-z
  y,-x+y,-z
  x-y,x,-z
  -y,-x,z
  -x+y,y,z
  x,x-y,z
  x+2/3,y+1/3,z+1/3
  -y+2/3,x-y+1/3,z+1/3
  -x+y+2/3,-x+1/3,z+1/3
  y+2/3,x+1/3,-z+1/3
  x-y+2/3,-y+1/3,-z+1/3
  -x+2/3,-x+y+1/3,-z+1/3
  -x+2/3,-y+1/3,-z+1/3
  y+2/3,-x+y+1/3,-z+1/3
  x-y+2/3,x+1/3,-z+1/3
  -y+2/3,-x+1/3,z+1/3
  -x+y+2/3,y+1/3,z+1/3
  x+2/3,x-y+1/3,z+1/3
  x+1/3,y+2/3,z+2/3
  -y+1/3,x-y+2/3,z+2/3
  -x+y+1/3,-x+2/3,z+2/3
  y+1/3,x+2/3,-z+2/3
  x-y+1/3,-y+2/3,-z+2/3
  -x+1/3,-x+y+2/3,-z+2/3
  -x+1/3,-y+2/3,-z+2/3
  y+1/3,-x+y+2/3,-z+2/3
  x-y+1/3,x+2/3,-z+2/3
  -y+1/3,-x+2/3,z+2/3
  -x+y+1/3,y+2/3,z+2/3
  x+1/3,x-y+2/3,z+2/3
```

```
_cell_length_a                    1.7023
_cell_length_b                    1.7023
_cell_length_c                    7.4994
_cell_angle_alpha                 90.0000
_cell_angle_beta                  90.0000
_cell_angle_gamma                 120.0000
loop_
_atom_site_label
_atom_site_type_symbol
_atom_site_fract_x
_atom_site_fract_y
_atom_site_fract_z
_atom_site_U_iso_or_equiv
_atom_site_adp_type
_atom_site_occupancy
O     O     1.33333    0.66667   -0.26134    0.00000   Uiso    1.00
loop_
_geom_bond_atom_site_label_1
_geom_bond_atom_site_label_2
_geom_bond_distance
_geom_bond_site_symmetry_2
_ccdc_geom_bond_type
O     O     1.080    16_544 S
```

Reference

[1] J. P. Perdew, K. Burke, and M. Ernzerhof, *Generalized Gradient Approximation Made Simple*, Phys. Rev. Lett. **77**, 3865 (1996).

[2] P. Strange, A. Svane, W. M. Temmerman, Z. Szotek, and H. Winter, *Understanding the Valency of Rare Earths from ®rst-Principles Theory*, **399**, 3 (1999).

[3] A. Togo and I. Tanaka, *First Principles Phonon Calculations in Materials Science*, Scr. Mater. **108**, 1 (2015).

[4] A. Carreras, A. Togo, and I. Tanaka, *DynaPhoPy: A Code for Extracting Phonon Quasiparticles from Molecular Dynamics Simulations*, Comput. Phys. Commun. **221**, 221 (2017).

[5] K. Schwarz, *DFT Calculations of Solids with LAPW and WIEN2k*, J. Solid State Chem. **176**, 319 (2003).

[6] K. Schwarz and P. Blaha, *Solid State Calculations Using WIEN2k*, Comput. Mater. Sci. **28**, 259 (2003).

[7] J. Heyd, G. E. Scuseria, and M. Ernzerhof, *Hybrid Functionals Based on a Screened Coulomb Potential*, J. Chem. Phys. **118**, 8207 (2003).

[8] J. Heyd, G. E. Scuseria, and M. Ernzerhof, *Erratum: "Hybrid Functionals Based on a Screened Coulomb Potential" [J. Chem. Phys. 118, 8207 (2003)]*, J. Chem. Phys. **124**, 219906 (2006).

[9] S. J. Clark, M. D. Segall, C. J. Pickard, P. J. Hasnip, M. I. J. Probert, K. Refson, and M. C. Payne, *First Principles Methods Using CASTEP*, Z. Für Krist. - Cryst. Mater. **220**, 567 (2005).

[10] K. Ohta, Y. Kuwayama, K. Hirose, K. Shimizu, and Y. Ohishi, *Experimental Determination of the Electrical Resistivity of Iron at Earth's Core Conditions*, Nature **534**, 95 (2016).

[11] Y. Zhang, M. Hou, G. Liu, C. Zhang, V. B. Prakapenka, E. Greenberg, Y. Fei, R. E. Cohen, and J.-F. Lin, *Reconciliation of Experiments and Theory on Transport Properties of Iron and the Geodynamo*, Phys. Rev. Lett. **125**, 078501 (2020).

[12] M. Pozzo and D. Alfè, *Saturation of Electrical Resistivity of Solid Iron at Earth's Core Conditions*, SpringerPlus **5**, 256 (2016).

[13] J. Xu, P. Zhang, K. Haule, J. Minar, S. Wimmer, H. Ebert, and R. E. Cohen, *Thermal Conductivity and Electrical Resistivity of Solid Iron at Earth's Core Conditions from First Principles*, Phys. Rev. Lett. **121**, 096601 (2018).

[14] F. D. Stacey and O. L. Anderson, *Electrical and Thermal Conductivities of Fe-Ni-Si Alloy under Core Conditions*, Phys. Earth Planet. Inter. **124**, 153 (2001).

[15] Y. Bi, H. Tan, and F. Q. Jing, *Electrical Conductivity of Iron under Shock Compression up to 200 GPa*, J. Phys.-Condens. Matter **14**, 10849 (2002).

[16] T. Guillot, *Interiors of Giant Planets inside and Outside the Solar System.*, Science (1999).
